# Effects of dietary *Hermetia illucens* meal inclusion on cecal microbiota and small intestinal mucin dynamics and infiltration with immune cells of weaned piglets

**DOI:** 10.1186/s40104-020-00466-x

**Published:** 2020-06-24

**Authors:** Ilaria Biasato, Ilario Ferrocino, Elena Colombino, Francesco Gai, Achille Schiavone, Luca Cocolin, Valeria Vincenti, Maria Teresa Capucchio, Laura Gasco

**Affiliations:** 1 Department of Agricultural, Forest and Food Sciences, University of Turin, Largo Paolo Braccini 2, 10095 Grugliasco, TO Italy; 2 Department of Veterinary Sciences, University of Turin, Largo Paolo Braccini 2, 10095 Grugliasco, TO Italy; 3 Institute of Science of Food Production, National Research Council, Largo Paolo Braccini 2, 10095 Grugliasco, TO Italy

**Keywords:** 16S rRNA, Gut health, *Hermetia illucens* L., Histology, Insect meal, Microbiota, Mucin, Pig

## Abstract

**Background:**

The constant interaction between diet and intestinal barrier has a crucial role in determining gut health in pigs. *Hermetia illucens* (HI) meal (that represents a promising, alternative feed ingredient for production animals) has recently been demonstrated to influence colonic microbiota, bacterial metabolite profile and mucosal immune status of pigs, but no data about modulation of gut mucin dynamics are currently available. The present study evaluated the effects of dietary HI meal inclusion on the small intestinal mucin composition of piglets, as well as providing insights into the cecal microbiota and the mucosal infiltration with immune cells.

**Results:**

A total of 48 weaned piglets were randomly allotted to 3 dietary treatments (control diet [C] and 5% or 10% HI meal inclusion [HI5 and HI10], with 4 replicate boxes/treatment and 4 animals/box) and slaughtered after 61 days of trial (3 animals/box, 12 piglets/diet). The cecal microbiota assessment by 16S rRNA amplicon based sequencing showed higher beta diversity in the piglets fed the HI-based diets than the C (*P* <  0.001). Furthermore, the HI-fed animals showed increased abundance of *Blautia*, *Chlamydia*, *Coprococcus*, *Eubacterium*, *Prevotella*, *Roseburia*, unclassified members of Ruminococcaceae, *Ruminococcus* and *Staphylococcus* when compared to the C group (FDR <  0.05). The gut of the piglets fed the HI-based diets showed greater neutral mucin percentage than the C (*P* <  0.05), with the intestinal neutral mucins of the HI-fed animals being also higher than the sialomucins and the sulfomucins found in the gut of the C group (*P* <  0.05). Furthermore, the piglets fed the HI-based diets displayed lower histological scores in the jejunum than the other gut segments (ileum [HI5] or ileum and duodenum [HI10], *P* <  0.05).

**Conclusions:**

Dietary HI meal utilization positively influenced the cecal microbiota and the small intestinal mucin dynamics of the piglets in terms of selection of potentially beneficial bacteria and preservation of mature mucin secretory architecture, without determining the development of gut inflammation. These findings further confirm the suitability of including insect meal in swine diets.

## Introduction

In the swine industry, which represents one of the major meat source for humans [1], the feed efficiency is a particularly critical aspect, since feed accounts for the majority of the total production costs [2]. Apart from animal genetics, disease, and production management, diet is considered one of the main factors influencing the feed efficiency in pigs [3]. The crucial role of the diet is related to its constant interaction with the gut barrier, which is constituted by microbiota and their products, mucus layers, host-derived antimicrobial compounds, epithelium, and underlying immune tissue [4]. In particular, researchers have focused their attention on the intestinal microbiota and mucin composition, as they can be widely affected by dietary modifications [5, 6]. The gut microbiota has a key impact on host metabolism, immune functions and physiology, thus exerting a significant influence on gut and systemic health, as well as nutrient processing and energy harvesting [7]. The intestinal commensal microbes also depend on diet and mucus for nutrient and energy source and binding sites, respectively [8]. Furthermore, the gut microbiota and microbial products are capable of modulating the mucin synthesis and secretion, both through the direct activation of several signalling cascades and the indirect generation of bioactive factors by the gut mucosa [6]. Mucins are multifunctional glycoproteins that compose the gut mucus layer and are mainly involved in the intestinal protection and nutrient digestion and absorption [9]. Therefore, investigating both the gut microbiota and the mucins seems to be fundamental in finding effective strategies for the improvement of pig intestinal health and feed efficiency, especially when a novel feed ingredient is tested. Another important aspect to consider is that piglets, especially in the postweaning period, are under great environmental pressure, thus causing a decline in their immune function and, in turn, development of gut inflammation. As a consequence, histological analysis of the gut may also provide useful information about the health status of the intestinal barrier [10].

Within the animal production scenario, the use of insects as alternative feed ingredients has rapidly become a consolidated reality, not only due to their remarkable nutritive properties and advantageous rearing characteristics [11], but also to their potential ability to modulate the intestinal microbiota with positive effects on animal health [12]. Among the insect species investigated for animal feeding purposes, *Hermetia illucens* (HI) has recently gained the greatest attention in pig farming [13–15]. In particular, HI prepupa and larva meals proved to be highly digestible and safe for weaned piglets, with no negative influence being observed on animal health and performance and gut mucosal morphology [16, 17]. These studies establish the bases of the authorization to feed the insect proteins to pigs, which is currently prohibited by the Regulation No 999/2001 [18]. Furthermore, significant *in vitro* gut antimicrobial effects against *D*-streptococci (opportunistic pathogens) have been ascribed to HI prepupa fat utilization, with the authors attributing these positive effects to its high content of lauric acid [16]. Yu et al. [19] recently reported that dietary HI larva meal inclusion may enhance the colonic mucosal immune homeostasis of finishing pigs via positively altering the bacterial composition and their metabolites, thus confirming the antimicrobial properties of HI previously highlighted. However, if the study of intestinal microbiota in insect-fed pigs has made significant progresses, data about gut mucin composition modulation by insect meal utilization are still lacking.

Based on the above reported background, the present study aims to evaluate the effects of dietary HI meal inclusion on gut microbiota, mucin composition and infiltration with immune cells of weaned piglets.

## Materials and methods

### Piglets and experimental design

The experimental design of the present study is reported by Biasato et al. [17]. In order to give a brief summary, 48 weaned piglets (20 ± 1 days of age, initial body weight: 6.1 ± 0.16 kg) were randomly distributed to four isoenergetic and isonitrogenous dietary treatments. Each diet was offered to 4 replicate pens (boxes) of 4 piglets each. Corn meal-, barley meal-, and soybean meal-based diet was used as the control diet (C), while the two experimental dietary treatments (indicated as HI5 and HI10) were obtained by including 5% and 10% partially defatted HI larva meal (Hermetia Baruth GmbHo. KG, Baruth / Mark, Germany), respectively, as partial replacements of the soybean meal. The chemical composition of the HI larva meal was as follows: 947.4 g/kg dry matter, 559 g/kg crude protein, and 85 g/kg ether extract, as fed. Details of the diets are shown in Table S1. The growth performance of the piglets were also evaluated throughout the experimental trial, as reported in details by Biasato et al. [17]. Briefly, no overall significant differences were observed for growth performance, except for the average daily feed intake of the second feeding phase showing a linear response to increasing HI larva meal levels. The experimental period lasted 61 days.

### Intestinal sampling and processing

A total of twelve piglets per treatment (three animals per box) were randomly selected and slaughtered in a commercial abattoir at the end of the experimental trial. The animals were stunned by electrocution and exsanguinated. Cecal content was collected into sterile plastic tubes that were promptly refrigerated (for a maximum of 2 h) and frozen at − 80 °C until DNA extraction. Intestinal segment samples (approximately 5 cm in length) of duodenum, jejunum and ileum were excised and flushed with 0.9% saline to remove all the content. The collected segments of intestine were the tract after the pylorus (duodenum), the mid jejunum (jejunum) and the tract before the ileocecal junction (ileum). Gut segments were fixed in 10% buffered formalin solution for histological examination and histochemical staining. Tissues were routinely embedded in paraffin wax blocks, sectioned at 5 μm thickness and mounted on glass slides.

### DNA extraction and sequencing

The nucleic acid was extracted by pooling the cecal content from three slaughtered piglets per box (four pools per feeding group). The total genomic DNA (gDNA) was extracted from the samples using the RNeasy Power Microbiome KIT (Qiagen, Milan, Italy) following the manufacturer’s instructions. One microliter of RNase (Illumina Inc., San Diego, CA) was added to digest RNA in the DNA samples with an incubation of 1 h at 37 °C. The DNA was quantified using the NanoDrop and standardized at 5 ng/μL. The gDNA was used to assess the microbiota by the amplification of the V3-V4 region of the 16S rRNA gene [20]. The PCR products were to the illumina metagenomic pipeline. Sequencing was performed with a MiSeq Illumina instrument (Illumina) with V3 chemistry and generated 250 bp paired-end reads, following the manufacturer’s instructions.

### Histochemical staining

The paraffin-embedded intestinal sections of the piglets were also submitted to a triple staining that demonstrated the different mucin subtypes, according to Rieger et al. [21]. Firstly, sections were stained with the periodic acid-Schiff, which identified the neutral mucins in magenta. The second staining step was the Alcian blue pH 2.5, which stained the sialomucins in turquoise. Finally, sections were stained with the high iron diamine, which identified the sulfomucins in brownish-purple to black [21].

### Mucin staining quantification

One slide per histochemical staining for each intestinal segment was examined by means of light microscopy. Five randomly selected high power fields per each slide were captured with a Nikon DS-Fi1 digital camera coupled to a Zeiss Axiophot microscope using a 20× objective lens and NIS-Elements F software was used for image capturing. Mucin staining quantification was then performed by Image®-Pro Plus software. The presence of mucins was estimated as the percentage of the gut mucosal area (covering both the crypts and the villi) that was positive for the histochemical staining, as previously described [22]. In particular, mucins were automatically identified by means of pixel classification [21].

### Histological examination

The paraffin-embedded intestinal sections of the piglets were submitted to the Haematoxylin & Eosin (HE) staining to evaluate the gut infiltration with immune cells, as reported in details by Biasato et al. [17]. One slide per HE section was examined by means of light microscopy. For each gut segment, the mucosa and the submucosa were separately assessed for the immune cell infiltrates (mucosa and submucosa) and the gut-associated lymphoid tissue (GALT) activation (submucosa) using a semiquantitative scoring system from 0 (absence of alterations) to 3 (severe alterations). The total score of each gut segment was then obtained by adding the mucosa and the submucosa scores.

### Bioinformatics and statistical analysis

Paired-end reads were first assembled with FLASH [23] and quality filtered (at Phred < Q20) using QIIME 1.9.0 software [24], and the recently described pipeline was adopted [25]. Briefly, Operational Taxonomic Units (OTUs) were picked at 97% of similarity and centroids sequences were used to assign taxonomy using the Greengenes 16S rRNA gene database (version 2013). Alpha diversity indices were calculated using the *diversity* function of the vegan package [26] and analyzed using the pairwise Wilcoxon rank sum test to assess the differences between the dietary treatments. A filtered OTU table was generated at 0.1% abundance in at least 2 samples through QIIME. The table was then used to build the Principal component analysis (PCA). OTU table displayed the highest taxonomy resolution. Weighted UniFrac distance matrices and OTU table were used to perform Adonis and ANOSIM statistical tests in R environment. Pairwise Kruskal-Wallis test was used to find significant differences in microbial taxa abundance among the dietary treatments. *P* values were adjusted for multiple testing and a false discovery rate (FDR) <  0.05 considered as significant.

The statistical analysis of the histochemical and the histological data was performed using IBM SPSS Statistics V20.0.0 software. In relation to the histochemical data, a generalized linear model (GLM) was fitted to allow the mean gut mucin staining percentages to depend on linear predictors such as diet, mucin type, intestinal segment and their corresponding interactions through a gamma probability distribution with a nonlinear link function (log). The piglet and the pen within treatment effect were also included in the GLM as the repeated factors. Differently, the histological data were tested by fitting a GLM that allowed the total gut scores to depend on linear predictors such as diet, intestinal segment and their interaction through a negative binomial response probability distribution with a nonlinear link function (log). The piglet and the pen within treatment effect were also herein included in the GLM as the repeated factors. A hybrid method for parameter estimation was used for both the GLMs and a type III analysis with Wald chi-square test was applied to assess the model effects. All the obtained results were expressed as least squares means and SEM and the interactions between the factor levels were evaluated by pairwise comparisons. *P* values < 0.05 were considered statistically significant.

## Results

### Cecal microbiota characterization

**Fig. 1:**
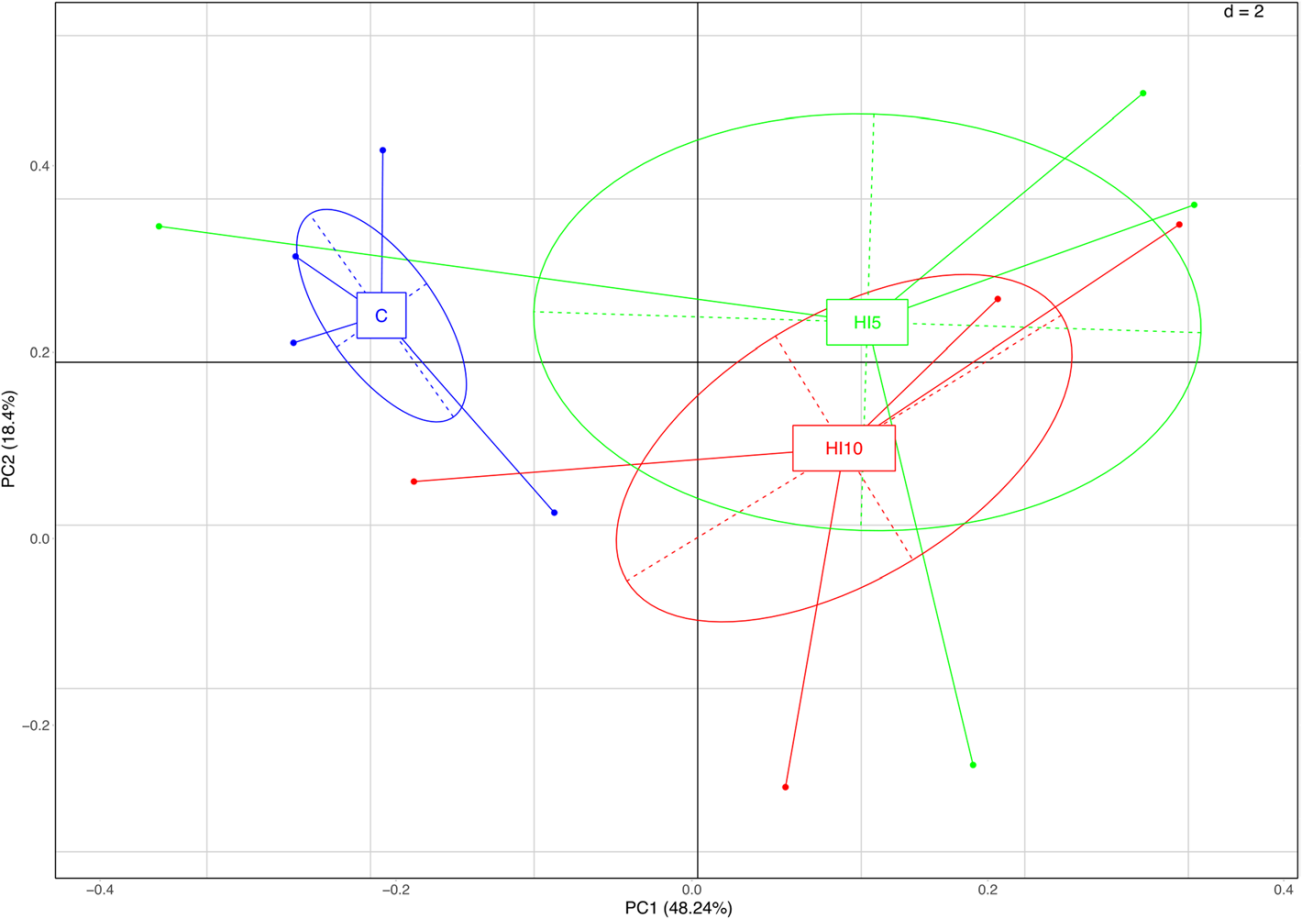
Bacterial community composition (weighted UniFrac beta diversity, PCA plots) in cecal samples of piglets fed control (C) and 5% (HI5) and 10% (HI10) inclusion levels of *Hermetia illucens* meal diets

A total of 916,380 raw reads (2× 250 bp) were obtained after sequencing. After joint and quality filtering, a total of 858,032 reads passed the filters applied through QIIME, with an average value of 71,502 reads/sample and a median sequence length of 465 bp. The rarefaction analysis and the Good’s coverage revealed a satisfactory coverage for all the samples (average Good’s coverage of 98%, Table S2). Dietary HI larva meal inclusion did not affect the alpha diversity indices (PD Whole Tree, Chao1, observed species richness and Shannon, Table S2, *P* > 0.05), whereas ADONIS and Anosim statistical tests based on Weighted UniFrac distance matrix showed significant differences among the dietary treatments (*P* < 0.001). In particular, the PCA showed a clear separation between the HI samples and those from the C-fed piglets (Fig. 1).

**Fig. 2:**
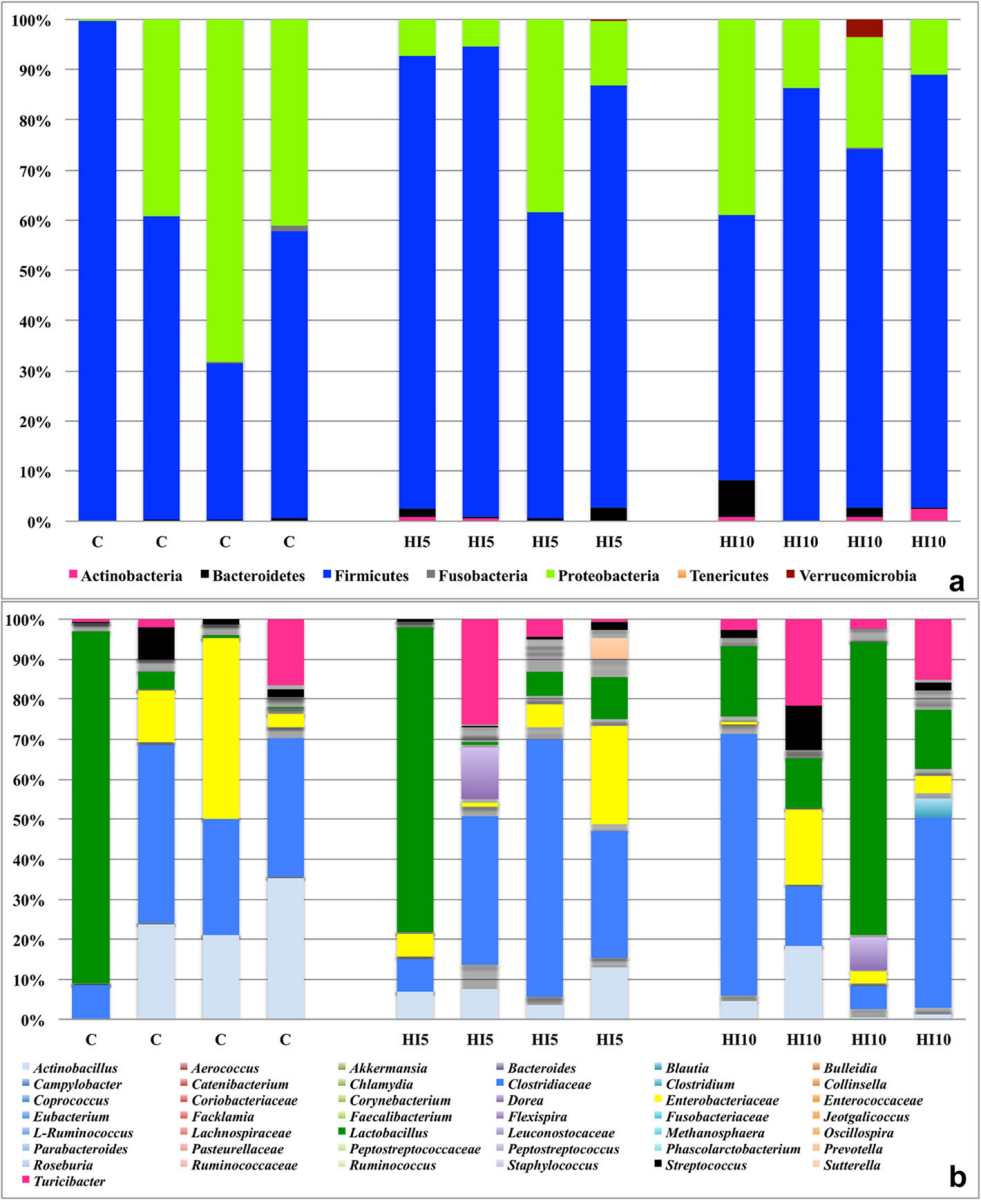
Relative abundance of the main bacterial phyla **(a)** and genera **(b)** in cecal samples of piglets fed control (C) and 5% (HI5) and 10% (HI10) inclusion levels of *Hermetia illucens* meal diets. Graph bar indicate the 4 replicate boxes per each dietary treatment

With regards to the most abundant OTUs, both the C- and the HI-fed groups showed Firmicutes, Proteobacteria and Bacteroidetes as predominant phyla in their cecal microbiota (Fig. 2, Table S3), as well as *Actinobacillus*, unclassified members (U. m.) of Clostridiaceae, U. m. of Enterobacteriaceae, *Lactobacillus* and *Streptococcus* as predominant genera (Fig. 2, Table S3).

**Fig. 3:**
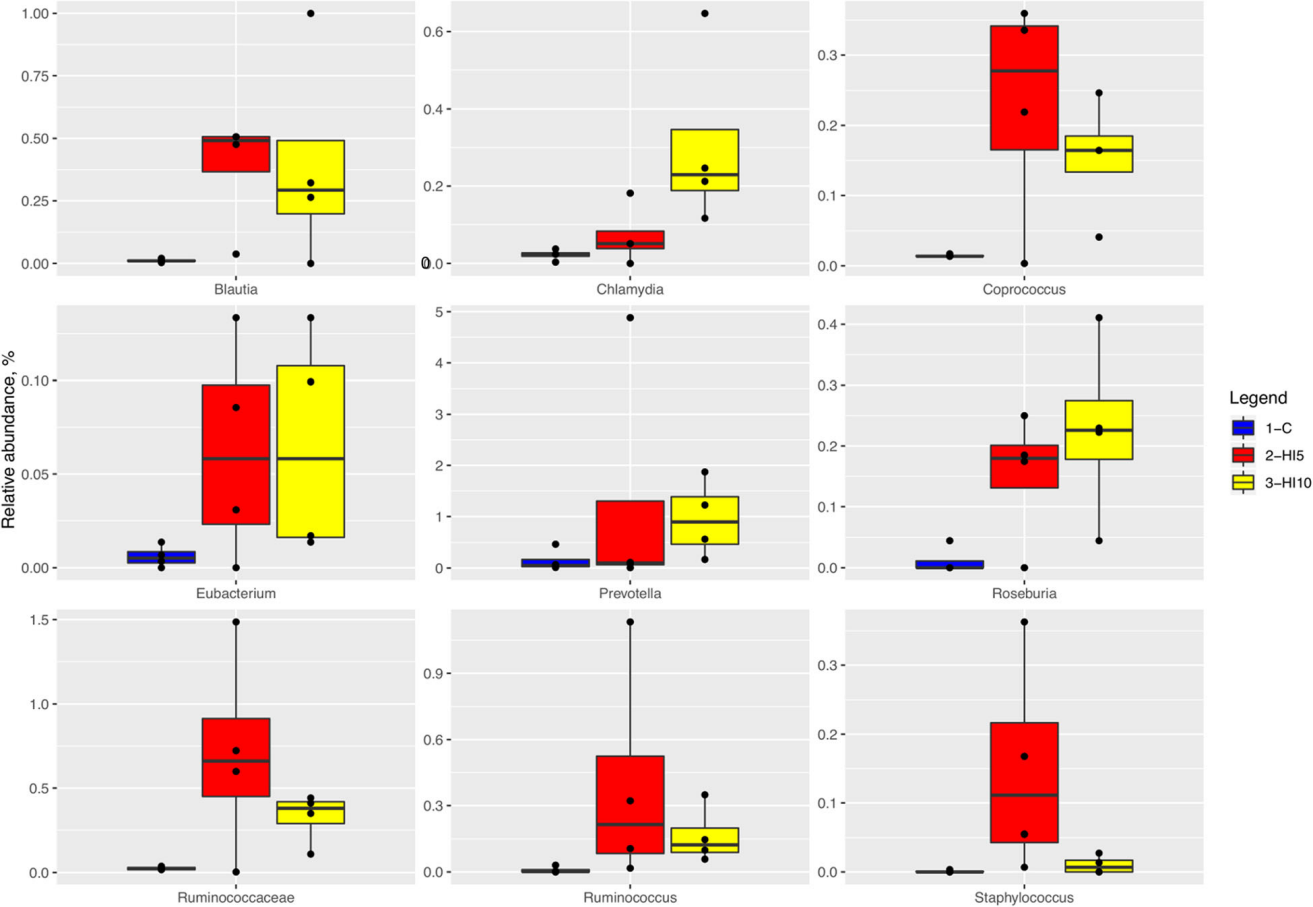
Relative abundance at phylum level of differentially abundant OTUs in cecal samples piglets fed control (C) and 5% (HI5) and 10% (HI10) inclusion levels of *Hermetia illucens* meal diets. Pairwise Kruskal-Wallis test, FDR < 0.05

Comparing the relative abundance of the main OTUs across the samples, the piglets fed the HI-based diets showed increased abundance of *Blautia*, *Chlamydia*, *Coprococcus*, *Eubacterium*, *Prevotella*, *Roseburia*, U. m. of Ruminococcaceae, *Ruminococcus* and *Staphylococcus* when compared to the C group (Fig. 3, FDR < 0.05).

### Intestinal mucin composition

**Fig. 4:**
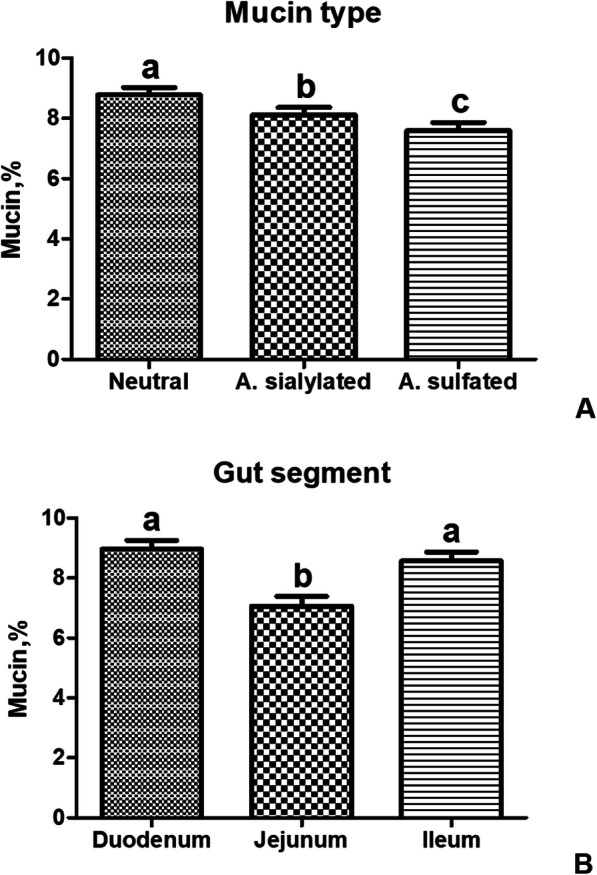
Gut mucin dynamics in the piglets of the present study. **(A)** Neutral, acidic sialylated (A. sialylated) and acidic sulfated (A. sulfated) mucin staining percentages in the small intestine independently of dietary insect meal inclusion. Graph bars with superscript letters (^a, b, c^) differ significantly (*P* < 0.05). **(B)** Duodenal, jejunal and ileal mucin staining percentages independently of dietary insect meal inclusion. Graph bars with superscript letters (a, b) differ significantly (*P* < 0.05). The mucin percentages are expressed as the percentage of the gut mucosal area (covering both the crypts and the villi) that was positive for the histochemical staining

**Fig. 5:**
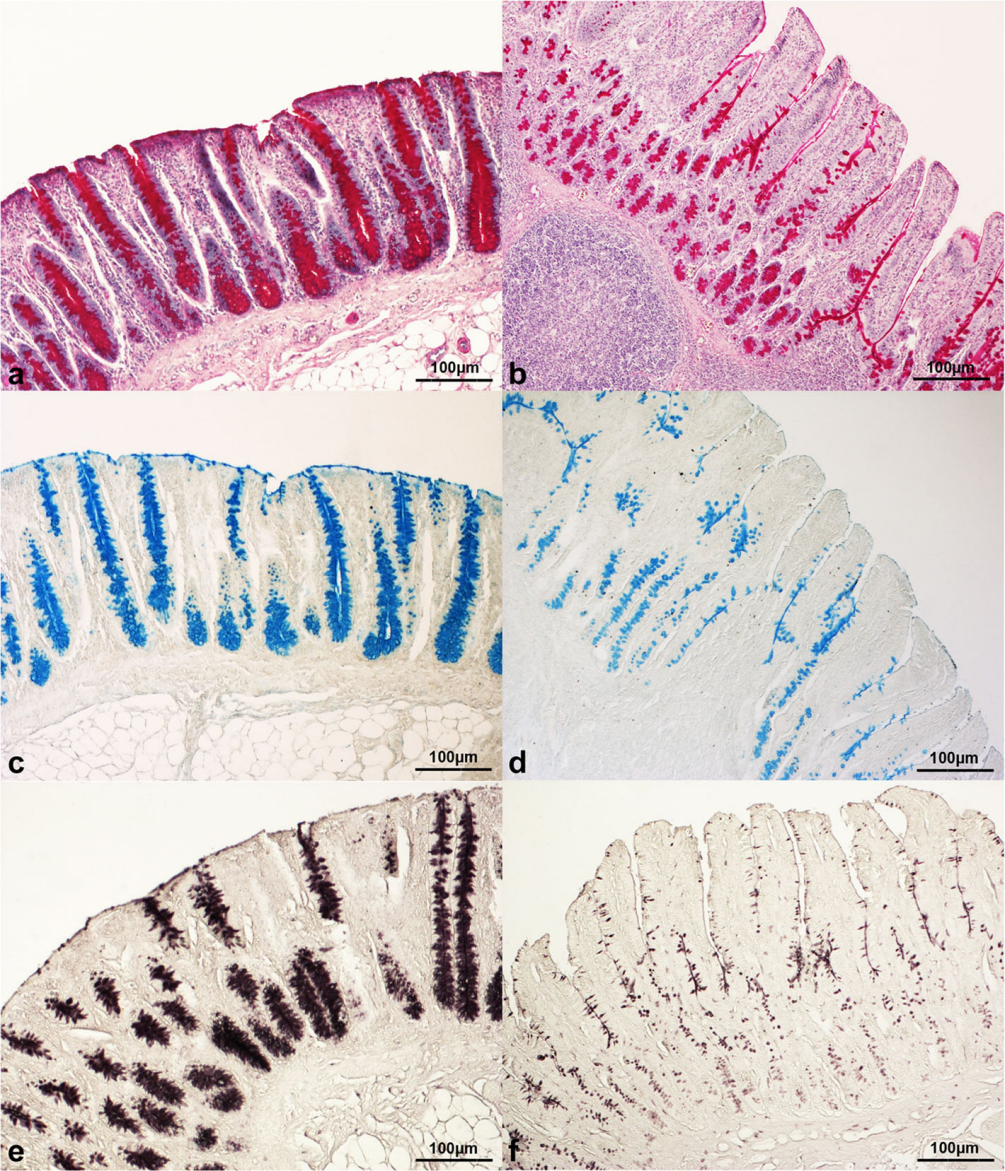
Histological pictures of ileal **(a, c, e)** and jejunal **(b, d, f)** samples stained with **(a, b)** periodic-acid Schiff (20× magnification), **(c, d)** Alcian Blue pH 2.5 (20× magnification) and **(e, f)** high iron diamine (20× magnification) from the piglets of the present study. Ileal samples show higher mucin staining intensity than the jejunal ones

**Fig. 6:**
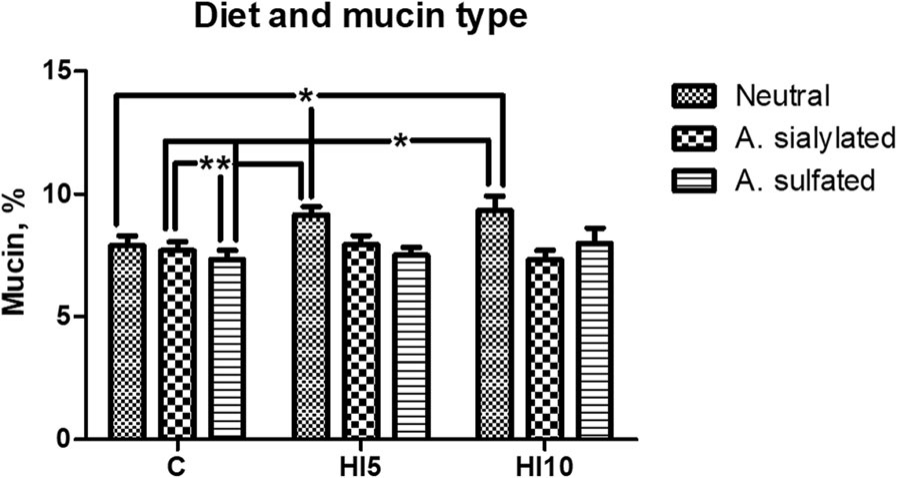
Neutral, acidic sialylated (A. sialylated) and acidic sulfated (A. sulfated) mucin staining percentages in the small intestine in relation to dietary insect meal inclusion. * = statistical significant (*P* < 0.05). The mucin percentages are expressed as the percentage of the gut mucosal area (covering both the crypts and the villi) that was positive for the histochemical staining

The mucin staining percentages in the gut of the piglets significantly depended on the mucin type (*P* < 0.001), the gut segment (*P* < 0.001), and interaction between the diet and the mucin type (*P* < 0.05). On the contrary, there was no significant influence of dietary HI meal inclusion (C = 7.64 ± 0.36; HI5 = 8.66 ± 0.58; HI10 = 8.17 ± 0.30) on the histochemical findings (*P* > 0.05). No significant interactions between the diet and the gut segment, the mucin type and the gut segment, and the diet, the gut segment and the mucin type (*P* < 0.05) were also identified (Table 1). In particular, the intestine showed higher neutral mucin staining percentage when compared to the other mucin subtypes (*P* < 0.001), with the sialomucins being also greater than the sulfomucins (*P* < 0.001, Fig. 4). Furthermore, higher mucin staining percentage was identified in the duodenum and the ileum in comparison with the jejunum (*P* < 0.001, Figs. 4 and 5). The gut of the HI-fed piglets also showed greater neutral mucin staining percentage than the C group (*P* < 0.05), with the intestinal neutral mucins of the HI animals being also higher than the sialomucins and the sulfomucins found in the gut of the C group (*P* < 0.05, Fig. 6).

**Table 1:** Effects of the different linear predictors on the histochemical findings and the histological scores in the gut of the piglets

Histochemical findings	d.f.^d^	Chi-square	*P*^f^
Diet^a^	2	2.614	0.271
Mucin type^b^	2	53.724	< 0.001
Gut segment^c^	2	34.164	< 0.001
Diet × Mucin type	4	12.216	0.016
Diet × Gut segment	4	1.017	0.907
Mucin type × Gut segment	4	7.878	0.096
Diet × Mucin type × Gut segment	8	11.507	0.175
Histological scores			
Diet	2	0.739	0.691
Gut segment	2	32.113	< 0.001
Diet × Gut segment	4	1.658	0.798

^a^Three dietary treatments: C = control; HI5 = 5% inclusion level of *Hermetia illucens*; HI10 = 10% inclusion level of *Hermetia illucens*

^b^Three types: neutral, acidic sialylated and acidic sulfated mucins

^c^Three gut segments: duodenum, jejunum and ileum

^d^Degrees of freedom

^f^Statistical significance: *P* < 0.05

### Intestinal infiltration with immune cells

**Fig. 7:**
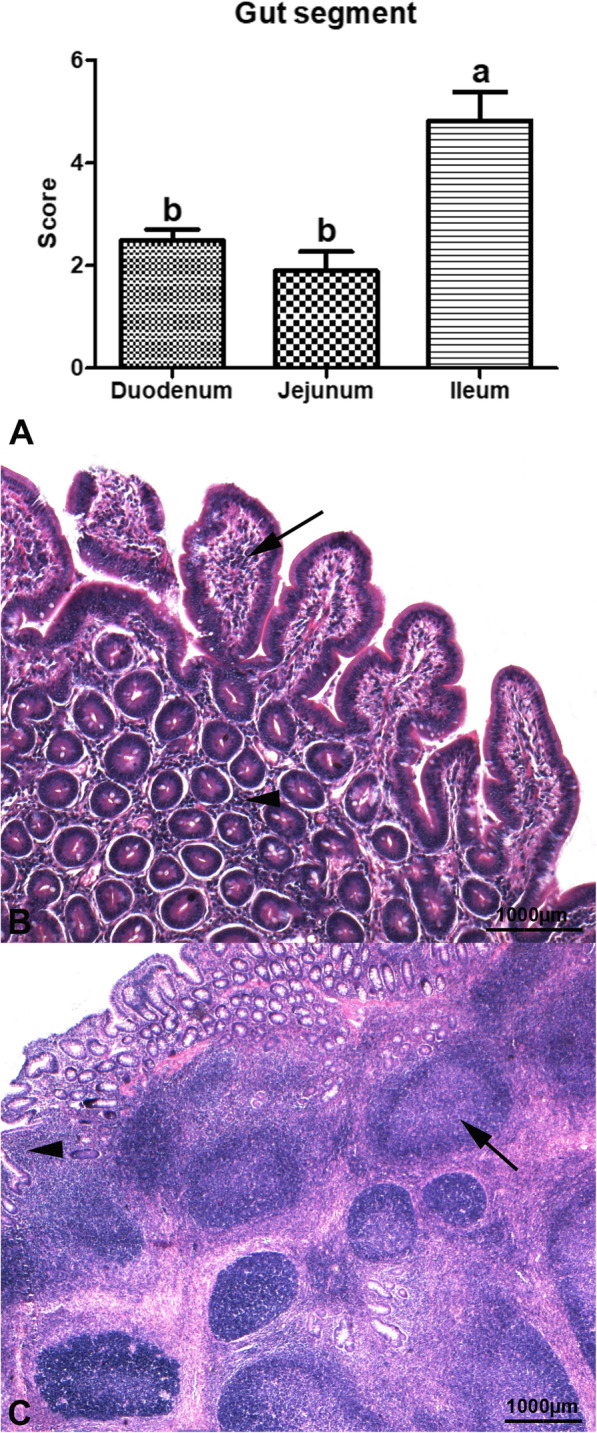
Gut infiltration with immune cells in the piglets of the present study. **(A)** Histological scores in the small intestine independently of dietary insect meal inclusion. Graph bars with superscript letters (a, b) differ significantly (*P* < 0.05). **(B)** HI5 group. Mild, multifocal mucosal (arrow) and submucosal (arrowhead) lymphoplasmacytic infiltration is observed in the jejunum. Haematoxylin & Eosin stain, 5× magnification). **(C)** C group. The ileum shows severe, focal mucosal and submucosal lymphoplasmacytic infiltration (arrowhead), as well as severe, multifocal Gut-Associated Lymphoid Tissue (GALT) activation (arrow). Haematoxylin & Eosin stain, 2.5× magnification

The histological scores in the gut of the piglets significantly depended on the gut segment (*P* < 0.001), while no significant influence of dietary HI meal inclusion (C = 2.66 ± 0.38; HI5 = 2.71 ± 0.32; HI10 = 3.16 ± 0.52) was observed (*P* > 0.05). The histological scores were also not significantly affected by the interaction between the diet and the gut segment (*P* > 0.05, Table 1). In particular, the ileum showed higher infiltration with immune cells when compared to the other gut segments (*P* < 0.001, Fig. 7).

## Discussion

### Cecal microbiota characterization

Firmicutes, Proteobacteria and Bacteroidetes represented the dominant bacterial phyla in both the C- and HI-fed piglets of the present study. These findings overall agree with the previous researches that identified Firmicutes [5, 27–29] as main bacterial phylum in the pig cecum, followed by Proteobacteria [27, 29] and Bacteroidetes [29]. In relation to the genera composition, *Actinobacillus*, *Lactobacillus* and *Streptococcus*, as well as members of Clostridiaceae and Enterobacteriaceae families, mainly colonized the cecal microbiota of the piglets fed either the C or the HI-based diets in the current research. These findings are also in agreement with the previous studies, which observed *Lactobacillus* [3, 5, 19, 27, 29], *Streptococcus* [5, 19, 27, 29] and *Actinobacillus* [29] as main bacterial genera in the cecal microbiota of pigs.

The microbial composition of the cecal digesta obtained from the piglets of the current research was significantly affected by dietary HI larva meal inclusion, as demonstrated by the increased beta diversity observed in the HI groups. This is in agreement with Yu et al. [19], who identified significant dissimilarities between the colonic microbiota of C- and HI-fed finishing pigs. These findings also confirm in swine species what previously reported in poultry, where insect meal utilization proved to be capable of creating a more diverse (and, in turn, stable) intestinal microbiota [25, 30, 31].

A specific signature at genus level was also observed in the cecal microbiota of the HI-fed piglets of the present study, where *Blautia*, *Chlamydia*, *Coprococcus*, *Eubacterium*, *Prevotella*, *Roseburia*, Ruminococcaceae, *Ruminococcus* and *Staphylococcus* were markedly predominant. *Blautia* [32], *Coprococcus* [33], *Eubacterium* [34], *Prevotella* [35–37], *Roseburia* [38], members of Ruminococcaceae [39, 40] and *Ruminococcus* [41, 42] are all taxon involved in polysaccharide degradation and fermentation, that boosted the production of short chain fatty acids (SCFAs) (mainly butyrate). The SCFAs have significant health benefits for the gut barrier [43], with butyrate being particularly essential for maintaining the intestinal metabolism [44], promoting the epithelial energy metabolism and stimulating the immune development [45]. *Prevotella* has also been reported to be involved in amino acid metabolism of host and positively influence the porcine intramuscular fat, which is considered an indicator for meat quality of pigs [37]. Furthermore, Ruminococcaceae family – that generally represents a core taxon with a relative abundance of 5% to 10% – is capable of improving the feed efficiency in pigs [3, 33]. Increase in SCFAs-producing bacteria, as well as SCFAs, has already been reported in both laying hens [30] and pigs [19] fed HI larva meal-based diets. These changes were attributed to the chitin content of the insect meal, which may serve as substrate for the gut microbiota, thus affecting either their composition or their microbial fermentation metabolites [19, 30]. Despite no SCFAs detection having been performed in the present study, the analogous identification of SCFAs-producing bacteria allows to hypothesize a similar way of action of HI larva meal in the piglets’ gut. Therefore, the increase in the above-mentioned bacterial taxa by HI larva meal utilization may have helped the piglets to maintain a healthy gut and show, consequently, similar growth performance to the C animals. Differently, in relation to the other increased OTUs observed in the HI-fed animals, *Chlamydia* and *Staphylococcus* genera comprises pathogenic bacteria, thus representing a potential negative finding. However, it is important to underline that the piglets fed the HI-based diets remained clinically healthy throughout the experimental trial and showed no significant alterations at the histological examination [17]. Since the growth performance were also overall unaffected by insect meal utilization, the positive increase in the SCFAs-producing bacteria could have mitigate this negative microbiota modulation.

### Intestinal mucin composition

Independently of dietary HI meal inclusion, the small intestine of the piglets of the present study showed higher neutral mucin staining percentage than the other subtypes. The physiologic relevance of the distinct mucin subtypes has not been well understood yet, with data in pigs being also particularly scarce and rather conflicting [46]. Increase in neutral mucins during post-weaning has been suggested to be related to the physiological variation in villi and crypt depth, thus, in turn, affecting the goblet cell differentiation and normal maturation [47]. Indeed, mucins in the neonatal piglets are highly acidic [48, 49]. Therefore, a predominance of neutral mucins is considered indicative of an increased intestinal maturity to facilitate the breakdown of complex carbohydrates [50]. As a confirmation of this aspect, Rieger et al. [21] recently observed higher staining percentage of neutral mucins (40%) than sulfomucins (8%) and sialomucins (2%) in weaned piglets from different feeding trials.

Independently of insect meal utilization, the small intestine of the piglets of the current research displayed greater mucin staining percentage in the duodenum and the ileum when compared to the jejunum. The mucin dynamics in the distinct segments of pig intestine have not been elucidated yet, since very limited studies have focused their attention on multiple intestinal tracts [21, 51]. However, each gut segment has its own specific characteristics that could explain the different histochemical findings. Indeed, the secretion of mucins in the duodenum has been related to the need of neutralizing of the acidic pH of the entering gastric juices [52]. Furthermore, since several pig pathogens (i.e., *Salmonella* Typhimurium and *Lawsonia intracellularis*) mainly colonize the ileal mucosa, the mucin production may be particularly useful as protective strategy. Therefore, the predominant staining of mucins observed in the duodenum and the ileum may be attributed to their different anatomy and physiology.

Interestingly, the piglets fed HI-based diets of the present study showed higher neutral mucin staining percentage in comparison with all the mucin subtypes of the C group. This could have positive implications, since the production of neutral mucins has been suggested to serve as a protective mechanism against invasion by pathogenic bacteria [53, 54]. Furthermore, as the identification of the neutral mucins is predominant in the mature gut, insect meal utilization may contribute to the preservation of a well-developed mucin secretory architecture. However, it is important to consider that both the gut sampling and the fixation methods may have caused the loss of most of the non-tissue mucins, thus representing a limitation of quantifying the gut mucins by histochemical analysis.

### Intestinal infiltration with immune cells

The present study provides a useful, easy-to-use, histological semiquantitative scoring system to give reliable information about the gut infiltration with immune cells in piglets. Biasato et al. [17] previously observed gut mucosal/submucosal lymphoplasmacytic or eosinophilic infiltrates – with or without GALT activation – in both the C- and the HI-fed piglets of the present study, attributing these alterations to the feeding practices and reporting no significant effect of insect meal utilization on the mean intestinal histological scores. The novel histological and statistical approaches herein adopted confirmed that dietary HI meal inclusion did not lead to the development of gut inflammation, but also revealed that the animals fed both the insect-based and the C diets of the current research displayed greater infiltration with immune cells in the ileum than the other gut segments. This is in agreement with the previously described mucin dynamics in the ileum, thus further underlying the predisposition of this gut segment to be colonized by potential pathogens and, consequently, recall immune cells as defense mechanism. Yu et al. [19] previously observed an up-regulation of the anti-inflammatory cytokines and intestinal barrier genes in HI-fed finishing pigs, attributing these changes to an increase in SCFAs-producing bacteria and their metabolites. The parallel identification of SCFAs-producing bacteria and infiltration of immune cells in the gut of the insect-fed piglets of the present study suggests the importance to perform both the histological examination and the gene expression analysis to characterize the intestinal inflammatory status.

## Conclusions

In conclusion, dietary HI meal inclusion up to 10% inclusion level may positively modulate the cecal microbiota (in terms of selection of SCFAs-producing bacteria) and the small intestinal mucin composition (in terms of stimulation of gut maturity) of the weaned piglets. Furthermore, the histological characterization of the gut infiltration with immune cells highlighted that insect meal utilization has not a significant role in its development. However, further researches performing gene expression analyses for gut mucin and cytokine characterization – as well as metagenomics and meta-metabolomics for the study of the microbiome – are needed to overcome the above-mentioned limitations and confirm the findings herein observed.

## Supplementary information

**Additional file 1: Table S1.** Ingredients and chemical composition of the experimental diets. cPremix: 16,000 IU vitamin A; 2,000 IU vitamin D_3_; 75.0 mg vitamin E; 2.94 mg vitamin K_3_; 3.0 mg vitamin B_1_; 6.0 mg vitamin B_2_; 4.0 mg vitamin B_6_; 0.05 mg vitamin B_12_; 98 mg vitamin C; 21.0 mg pantothenic acid; 40.0 mg vitamin PP; 1.20 mg folic acid; 0.25 mg biotin; 1500 UI 6-phytase; 700 UI xylanase; 312.5 UI glucanase; 145.68 mg copper; 0.05 mg cobalt; 0.44 mg selenium. HI, *Hermetia illucens*; DM, dry matter; CP, crude protein; EE, ether extract; NDF, neutral detergent fiber; ADF, acid detergent fiber; NE, net energy; C = control; HI5 = 5% inclusion level of *Hermetia illucens*; HI10 = 10% inclusion level of *Hermetia illucens*.

**Additional file 2: Table S2.** Good’s coverage and α-diversity measures of cecal microbiota of piglets fed control (C) and 5% (HI5) and 10% (HI10) inclusion level of *Hermetia illucens* meal diets. Description column indicates the 4 replicate boxes per each dietary treatment.

**Additional file 3: Table S3.** Relative abundance of the main bacterial phyla and genera of cecal microbiota of piglets fed control (C) and 5% (HI5) and 10% (HI10) inclusion levels of *Hermetia illucens* meal diets.

## Data Availability

The datasets analysed in the present study are available from the corresponding author on reasonable request.

## Abbreviations

CP: Crude protein
DM: Dry matter
EE: Ether extract
FDR: False discovery rate
GALT: Gut-associated lymphoid tissue
gDNA: Genomic DNA
HI: *Hermetia illucens*
NE: Net energy
OTU: Operational taxonomic unit
PCA: Principal component analysis
SEM: Standard error of the mean
SCFAs: Short chain fatty acids
